# EEG Signals Index a Global Signature of Arousal Embedded in Neuronal Population Recordings

**DOI:** 10.1523/ENEURO.0012-22.2022

**Published:** 2022-06-07

**Authors:** Richard Johnston, Adam C. Snyder, Rachel S. Schibler, Matthew A. Smith

**Affiliations:** 1 Department of Biomedical Engineering, Carnegie Mellon University, Pittsburgh, PA 15213; 2 Neuroscience Institute, Carnegie Mellon University, Pittsburgh, PA 15213; 3 Center for the Neural Basis of Cognition, Carnegie Mellon University, Pittsburgh, PA 15213; 4 Department of Brain and Cognitive Sciences, University of Rochester, Rochester, PA 14627; 5 Department of Neuroscience, University of Rochester, Rochester, NY 14642; 6 Center for Visual Science, University of Rochester, Rochester, NY 14627; 7 Harvey Mudd College, Claremont, CA 91711

**Keywords:** α, electroencephalography, microsaccade, pupil, slow drift

## Abstract

Electroencephalography (EEG) has long been used to index brain states, from early studies describing activity in the presence and absence of visual stimulation to modern work employing complex perceptual tasks. These studies have shed light on brain-wide signals but often lack explanatory power at the single neuron level. Similarly, single neuron recordings can suffer from an inability to measure brain-wide signals accessible using EEG. Here, we combined these techniques while monkeys performed a change detection task and discovered a novel link between spontaneous EEG activity and a neural signal embedded in the spiking responses of neuronal populations. This “slow drift” was associated with fluctuations in the subjects’ arousal levels over time: decreases in prestimulus α power were accompanied by increases in pupil size and decreases in microsaccade rate. These results show that brain-wide EEG signals can be used to index modes of activity present in single neuron recordings, that in turn reflect global changes in brain state that influence perception and behavior.

## Visual Abstract

**Figure:**
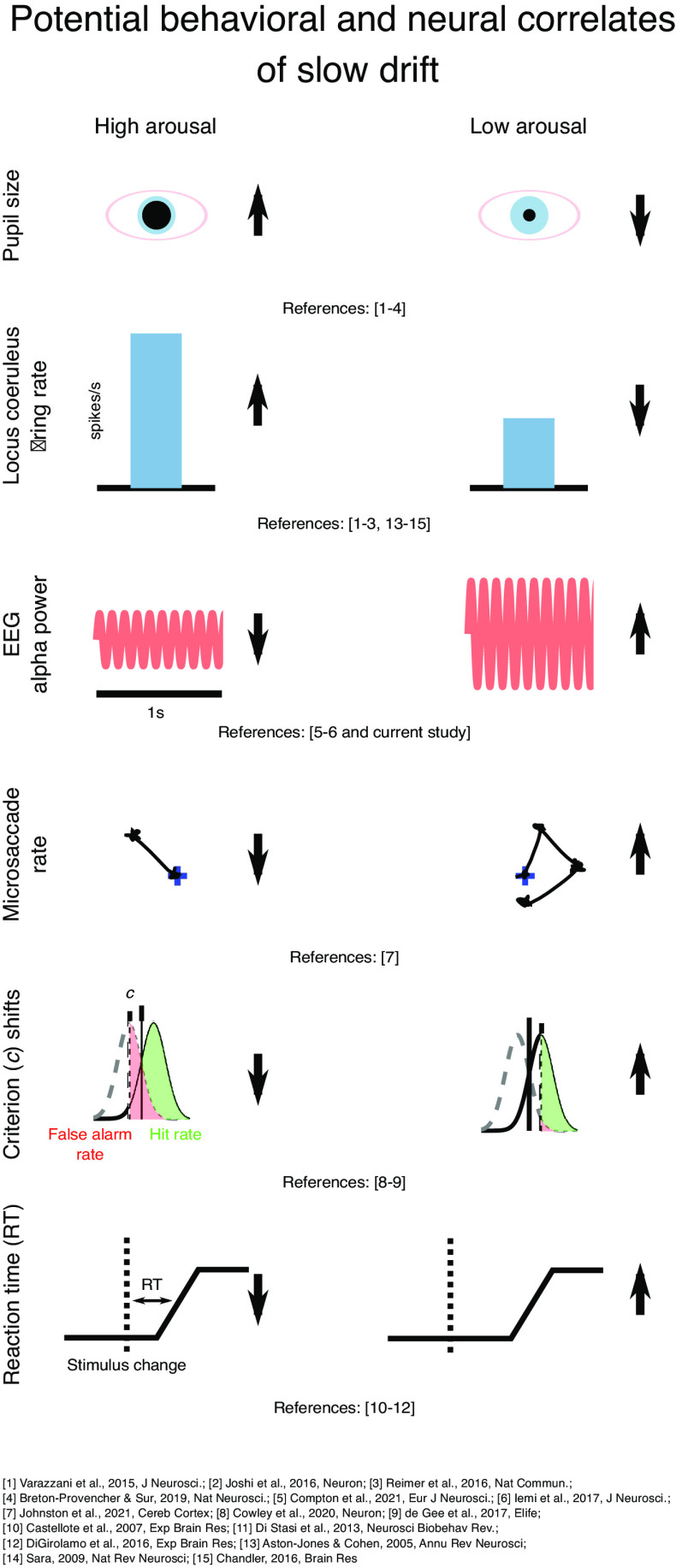

## Significance Statement

Decades of research has used electroencephalography (EEG) to investigate how voltage fluctuations on the scalp are related to cognition. These studies are useful for measuring brain-wide signals in a noninvasive manner, but they lack the ability to detect small-scale changes at the level of single neurons. In this study, we bridged this gap by recording EEG and spiking responses in the brain while macaques performed a perceptual decision-making task. We found that a commonly used metric of arousal in human EEG studies, prestimulus α power, is associated with slow drifts in the activity of cortical neurons. Together, these recordings made noninvasively on the scalp and directly in the brain were predictive of changes in arousal levels over time.

## Introduction

For decades, researchers have investigated how the spiking responses of single cortical neurons relate to performance on decision-making (Britten et al., 1996), attention (Moran and Desimone, 1985) and working memory (Fuster and Alexander, 1971) tasks. Interactions between pairs of neurons have also been studied extensively since technological advances in neural recording systems (e.g., microelectrode arrays and two-photon imaging) made it possible to monitor the activity of neural populations simultaneously (Zohary et al., 1994; Cohen and Maunsell, 2009; Leavitt et al., 2017). At the same time, it is becoming increasingly apparent that major insight about the neurobiological basis of cognition can be gained from the study of populations of neurons (Cohen and Maunsell, 2011; Harvey et al., 2012; Mante et al., 2013; Driscoll et al., 2017; Murray et al., 2017; Ni et al., 2018; Remington et al., 2018; Khanna et al., 2019; Oby et al., 2019; Valente et al., 2021). Furthermore, it has been shown that low-dimensional neural activity patterns can be used to index global brain states, which influence performance on cognitive tasks. For example, Stringer et al. (2019) applied principal component analysis (PCA) to data recorded from >10,000 neurons in the mouse and found that fluctuations in the first principal component were associated with a host of arousal-related variables including whisking, pupil size, and running speed. Musall et al. (2019) found that uninstructed movements, which themselves may occur at varying frequency based on arousal, were related to brain-wide activity in the mouse. In our own work in rhesus macaques, we have reported a pervasive “slow drift” of neural activity (Cowley et al., 2020), which is correlated with a distinctive pattern of eye metrics that is strongly indicative of changes in arousal (Johnston et al., 2021). However, it is unknown whether slow drift is associated with other arousal-related variables that can be measured in a rapid, accurate and noninvasive manner.

Spontaneous (i.e., prestimulus) oscillations in the α frequency band (∼8–12 Hz) are associated with lateralized changes in spatial attention and global changes in arousal. For example, studies investigating the effects of spatial attention on electroencephalography (EEG) activity have found that prestimulus α power is decreased in the visual cortex contralateral to the attended location (Worden et al., 2000; Sauseng et al., 2005; Kelly et al., 2006; Thut et al., 2006). In contrast, studies exploring the processes underlying perceptual decision-making have uncovered an association between task performance and brain-wide changes in prestimulus α power. To be more precise, the likelihood of detecting a near threshold visual stimulus increases when prestimulus oscillations in the α band decrease (Ergenoglu et al., 2004; Babiloni et al., 2006; Hanslmayr et al., 2007; van Dijk et al., 2008; Busch et al., 2009; Mathewson et al., 2009; Romei et al., 2010). Recent work suggests that these global effects (that occur across a range of frontal, midline, and occipital sites) arise because of changes in arousal. According to signal detection theory (Green and Swets, 1966), differences in performance on perceptual decision-making tasks can either reflect shifts in sensitivity or response criterion. Several studies have sought to dissociate these components in macaque monkeys (Luo and Maunsell, 2015, 2018; Crapse et al., 2018; Jun et al., 2021), and similar work has been conducted in human subjects using EEG. For example, it has been shown that brain-wide decreases in prestimulus α power are associated with increased hit rate and false alarm rate on perceptual decision-making tasks (Limbach and Corballis, 2016; Iemi et al., 2017). These results point to a link between prestimulus α power and response criterion, a variable that is modulated, at least in part, by subcortical regions that control arousal levels (de Gee et al., 2017).

One structure that has been implicated in the control of arousal is the locus coeruleus (LC; Aston-Jones and Cohen, 2005; Sara, 2009; Chandler, 2016). This small region in the pons represents the primary source of norepinephrine to the central nervous system and drives fluctuations in raw and evoked (baseline-corrected) pupil size: noninvasive markers that have been used extensively in the neurosciences to index changes in arousal (Varazzani et al., 2015; Joshi et al., 2016; Reimer et al., 2016; Breton-Provencher and Sur, 2019). Given that the LC is involved in modulating response criterion (de Gee et al., 2017) and pupil size (Joshi and Gold, 2020), one might also expect it to exert an influence on prestimulus α power. To test this hypothesis noninvasively, one could determine whether there is a significant association between prestimulus α power and pupil size. Several studies have used a combination of EEG and pupillometry to explore whether this is the case in humans (Hong et al., 2014; Van Kempen et al., 2019; Podvalny et al., 2021). For example, Compton et al. (2021) had subjects perform a classic Stroop task and found that α power during intertrial periods was inversely related to raw pupil size. That is, trials with greater pupil size were associated with reduced power in the α band and vice versa. These results suggest that spontaneous EEG signals can be used to index global brain state and raise the possibility that they might be associated with other arousal-related metrics such as microsaccade rate.

Microsaccades are small eye movements that occur at a rate of 1–2 Hz through the activity of neurons in the superior colliculus (SC; Rolfs, 2009). As with larger saccades (Burr et al., 1994; Diamond et al., 2000; Knöll et al., 2011), research has shown that visual perception is altered in the hundreds of milliseconds following a microsaccade (Hafed and Krauzlis, 2010; Hafed, 2013; Chen et al., 2015; Chen and Hafed, 2017; Scholes et al., 2018). Interestingly, Bellet et al. (2017) found that these short timescale modulations occur in a rhythmic manner at a frequency of 8–20 Hz. However, it is unclear whether a relationship exists between α oscillations and fixational eye movements at longer timescales, which are more likely to be associated with changes in a subject’s internal state (Cowley et al., 2020). Recent work in our laboratory found that slow fluctuations in raw pupil size over the course of a recording session were negatively correlated with microsaccade rate (Johnston et al., 2021). That is, microsaccade rate decreased under conditions of heightened arousal (as indexed by greater pupil size) and vice versa. As described above, the relationship between prestimulus α power and microsaccade rate has not yet been explored at long timescales. However, based on our previous results, one might expect there to be a positive correlation between these two variables over time.

**Figure 1.**
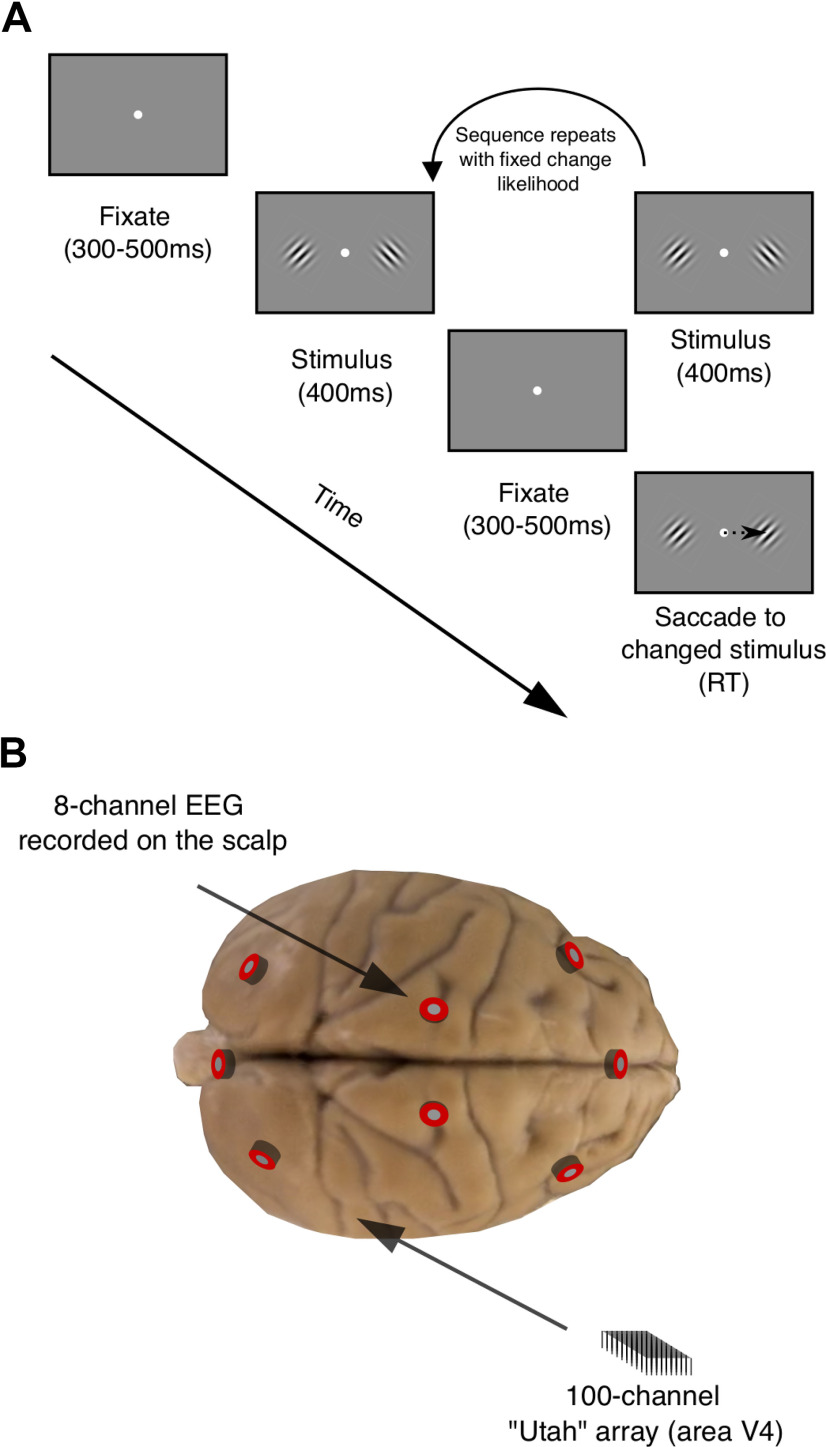
Experimental methods. ***A***, Orientation-change detection task. After an initial fixation period, a sequence of stimuli (orientated Gabor pairs separated by brief prestimulus periods spanning 300–500 ms) was presented. The subject’s task was to detect an orientation change in one of the stimuli and make a saccade to the changed stimulus. ***B***, Electrophysiological recordings. We simultaneously recorded: (1) spiking responses of populations of neurons in V4 using 100-channel microelectrode (“Utah”) arrays; and (2) EEG from eight electrodes positioned on the scalp. Spiking responses of populations of neurons in V4 were recorded during 400-ms stimulus periods, whereas EEG was recorded during the first 300 ms of prestimulus periods. RT, reaction time.

The primary aim of this study was to determine whether EEG signals recorded on the scalp can be used to index a neural measure of brain state acquired directly from the spiking activity of neural populations termed “slow drift.” In addition, we investigated whether prestimulus oscillations in the α band are associated with two noninvasive metrics that have previously been used to index global shifts in arousal and that we have found to be related to “slow drift” (Johnston et al., 2021): raw pupil size and microsaccade rate. EEG from the scalp and spiking activity from populations of neurons in V4 was simultaneously recorded from two monkeys while they performed an orientation-change detection task (Fig. 1*A*). Results showed that fluctuations in prestimulus α power over the course of a recording session were companied by changes in raw pupil size and microsaccade rate. As expected, prestimulus power in the α band was negatively correlated with raw pupil size and positively correlated with microsaccade rate. Interestingly, we also found a significant correlation between prestimulus α power and neural slow drift. This finding is of particular importance as it suggests that spontaneous components of the EEG signal recorded noninvasively on the scalp index low-dimensional patterns of neural activity acquired from microelectrode array recordings in the brain. These results support previous research showing that slow drift is associated with changes in arousal over time (Cowley et al., 2020; Johnston et al., 2021), and provide a strong link between global measurements made across recording modalities and species.

## Materials and Methods

### Subjects

Two adult male rhesus macaque monkeys (*Macaca mulatta*) were used in this study. A previous report (Snyder et al., 2018b) presented analysis of different aspects of the same experiments described here. This study reports the results from a subset of the data from Snyder et al. (2018b) in which EEG was recorded. Surgical procedures to chronically implant a titanium head post (to immobilize the subjects’ heads during experiments) and microelectrode arrays were conducted in aseptic conditions under isoflurane anesthesia, as described in detail by Smith and Sommer (2013). Opiate analgesics were used to minimize pain and discomfort during the perioperative period. Neural activity was recorded using 100-channel “Utah” arrays (Blackrock Microsystems) in V4 (Monkey Pe = right hemisphere; Monkey Wa = left hemisphere). The arrays comprised a 10 × 10 grid of silicon microelectrodes (1 mm in length) spaced 400 μm apart. Experimental procedures were approved by the Institutional Animal Care and Use Committee of the University of Pittsburgh and were performed in accordance with the United States National Research Council’s *Guide for the Care and Use of Laboratory Animals*.

### Microelectrode array recordings

Signals from each microelectrode in the array were amplified and bandpass filtered (0.3–7500 Hz) by a Grapevine system (Ripple). Waveform segments crossing a threshold (set as a multiple of the root mean square noise on each channel) were digitized (30 kHz) and stored for offline analysis and sorting. First, waveforms were automatically sorted using a competitive mixture decomposition method (Shoham et al., 2003). They were then manually refined using custom time amplitude window discrimination software (code available at https://github.com/smithlabvision/spikesort), which takes into account metrics including (but not limited to) waveform shape and the distribution of interspike intervals (Kelly et al., 2007). A mixture of single and multiunit activity was recorded, but we refer here to all units as “neurons.” The mean number of V4 neurons across sessions was 41 (SD = 10) for Monkey Pe and 21 (SD = 10) for Monkey Wa.

### EEG recordings

We recorded EEG from eight Ag/AgCl electrodes (Grass Technologies) adhered to the scalp with electrically conductive paste. The electrodes were positioned roughly at the following locations: Fz, Iz, CP3, CP4, F5, F6, PO7, and PO8 (Fig. 1*B*). Signals for each electrode were referenced online to a steel screw on the titanium head post, digitized at 1 kHz and amplified by a Grapevine system (Ripple) and low-pass filtered online at 250 Hz. They were then rereferenced to the average activity across all electrodes for the entire session. One of the challenges associated with simultaneously recording EEG and the spiking responses of neural populations is the introduction of craniotomies and microelectrode recording arrays. Importantly, previous work in our lab has shown that this does not significantly alter the way current flows to the scalp (Snyder et al., 2018a). We found that FFTs computed before and after craniotomies (performed to implant microelectrode arrays) are highly correlated suggesting that our results are generalizable to EEG recorded from human subjects with an intact skull. Segments of EEG data were recorded for each electrode during the first 300 ms of prestimulus periods on the change detection task (Fig. 1*A*). A constant duration was needed to remove aperiodic activity that has a 1/f-like distribution (Donoghue et al., 2020). We did not include the first prestimulus period because of an increase in eye position variability resulting from fixation having been established a short time earlier. Such variability was not present in the following prestimulus periods (see Johnston et al., 2021, their Fig. 1). Several outlier rejection steps were then taken. First, segments of EEG data were considered excessively noisy and removed if any of the electrodes had a SD that was 10 times greater than the mean of the entire session, or if any of the electrodes exhibited a flat signal defined as a SD <300 nanovolts (Snyder et al., 2018a). Segments of EEG data were also removed if there was evidence of excessive variability in the eye trace. For each session, we computed 1D eye velocity during each prestimulus period. EEG segments were removed if the SD of the eye velocity was two times greater than the mean eye velocity across all prestimulus periods. This final step was necessary to ensure that changes in prestimulus α power did not arise because of eye movement artifacts.

### Visual stimuli

Visual stimuli were generated using a combination of custom software written in MATLAB (The MathWorks) and Psychophysics Toolbox extensions (Brainard, 1997; Pelli, 1997). They were displayed on a CRT monitor (resolution = 1024 × 768 pixels; refresh rate = 100 Hz), which was viewed at a distance of 36 cm and γ-corrected to linearize the relationship between input voltage and output luminance using a photometer and look-up-tables.

### Behavioral task

Subjects fixated a central point (diameter = 0.6°) on the monitor to initiate a trial (Fig. 1*A*). Each trial comprised a sequence of stimulus periods (400 ms) separated by brief prestimulus (fixation) periods. The duration of each prestimulus period was drawn at random from a uniform distribution spanning 300–500 ms, but EEG data were only analyzed during the first 300 ms (see above). The 400-ms stimulus periods comprised pairs of drifting full-contrast Gabor stimuli. One stimulus was presented in the aggregate receptive field (RF) of the recorded V4 neurons, whereas the other stimulus was presented in the mirror-symmetric location in the opposite hemifield. Although the spatial (Monkey Pe = 0.85 cycles/°; Monkey Wa = 0.85 cycles/°) and temporal frequencies (Monkey Pe = 8 cycles/s; Monkey Wa = 7 cycles/s) of the stimuli were not optimized for each individual V4 neuron they did evoke a strong response from the population. The orientation of the stimulus in the aggregate RF was chosen at random to be 45° or 135°, and the stimulus in the opposite hemifield was assigned the other orientation. There was a fixed probability (Monkey Pe = 30%; Monkey Wa = 40%) that one of the Gabors would change orientation by ±1°, ±3°, ±6°, or ±15° on each stimulus presentation. The sequence continued until the subject: (1) made a saccade to the changed stimulus within 400 ms (“hit”); (2) made a saccade to an unchanged stimulus (“false alarm”); or (3) remained fixating for >400 ms after a change occurred (“miss”). If the subject correctly detected an orientation change, they received a liquid reward. In contrast, a time-out occurred if the subject made a saccade to an unchanged stimulus delaying the beginning of the next trial by 1 s. It is important to note that the effects of spatial attention were also investigated (although not analyzed in this study) by cueing blocks of trials such that the orientation change was 90% more likely to occur in one hemifield relative to the other hemifield.

### Eye tracking

Eye position and pupil diameter were recorded monocularly at a rate of 1000 Hz using an infrared eye tracker (EyeLink 1000, SR Research).

### Microsaccade detection

Microsaccades were defined as eye movements that exceeded a velocity threshold of 6 times the SD of the median velocity for at least 6 ms (Engbert and Kliegl, 2003). They were required to be separated in time by at least 100 ms. In addition, we removed microsaccades with an amplitude >1° and a velocity >100°/s. To assess the validity of our microsaccade detection method, the correlation (Pearson product-moment correlation coefficient) between the amplitude and peak velocity of detected microsaccades (i.e., the main sequence) was computed for each session. The mean correlation between these two metrics across sessions was 0.84 (SD = 0.06) indicating that our detection algorithm was robust as microsaccades fell on the main sequence (Zuber et al., 1965).

### Prestimulus power

For each electrode, FFTs were computed using Hanning-windowed segments of EEG data spanning the first 300 ms of the prestimulus period (Fig. 1*A*). Note that we did not include the first prestimulus period because of an increase in eye position variability resulting from fixation having been established a short time earlier (see above). The data for each electrode were then binned using a 30-min sliding window (step size = 6 min), which yielded eight FFTs per time bin (one for each electrode). We wanted to rule out the possibility that slow drift was associated with gradual changes in 1/f noise (Donoghue et al., 2021). Therefore, the aperiodic component of the signal was estimated by fitting an exponential to the binned FFTs for each electrode (Donoghue et al., 2020). Residual power was then computed by subtracting off the aperiodic portion of the signal. Previous research has shown that prestimulus α power is associated with performance on visual detection tasks across a range of frontal, midline, and posterior electrodes (Ergenoglu et al., 2004; Busch et al., 2009; Iemi et al., 2017). Hence, the aperiodic adjusted (residual) FFTs from all eight electrodes were averaged together for each time bin. Finally, we computed the mean residual power in different frequency bands. For each time bin, residual power was computed in the θ (4–8 Hz), α (8–12 Hz), β (12–30 Hz), and γ (30–50 Hz) bands.

### Eye metrics

Mean pupil diameter was measured during stimulus periods, whereas microsaccade rate was measured during prestimulus periods (Johnston et al., 2021). We did not include the initial fixation period when measuring microsaccade rate. As described above, there was an increase in eye position variability during this period resulting from fixation having been established a short time earlier (300–500 ms). Such variability was not present in following prestimulus periods (see Johnston et al., 2021, their Fig. 1). Reaction time and saccade velocity were measured on trials in which the subjects were rewarded for correctly detected an orientation change. Reaction time was defined as the time from when the change occurred to the time at which the saccade exceeded a velocity threshold of 150°/s. Saccade velocity was the peak velocity of the saccade to the changed stimulus. To isolate slow changes in the eye metrics over time, the data for each session was binned using a 30-min sliding window stepped every 6 min. The width of the window, and the step size, were chosen to isolate slow changes over time based on previous research. They were the same as those used by Cowley et al. (2020) and Johnston et al. (2021), which meant direct comparisons could be made across studies.

### Calculating slow drift

The spiking responses of populations of neurons in V4 were measured during a 400-ms period that began 50 ms after stimulus presentation (Fig. 1*A*). Research has shown that neurons in V4 are tuned for stimulus orientation (Desimone and Schein, 1987). To prevent the PCA identifying components related to stimulus tuning, residual spike counts were computed by subtracting the mean response for a given orientation (45° or 135°) across the entire session from individual responses to that orientation. To isolate slow changes in neural activity over time, residual spike counts for each V4 neuron were binned using a 30-min sliding window stepped every 6 min. PCA was then performed to reduce the high-dimensional residual data to a smaller number of latent variables (Cunningham and Yu, 2014). Slow drift in V4 was estimated by projecting the binned residual spike counts for each neuron along the first principal component.

### Aligning slow drift across sessions

As described above, slow drift was calculated by projecting binned residual spike counts along the first principal component. The weights in a PCA can be positive or negative (Jolliffe and Cadima, 2016), which meant the sign of the correlation between slow drift and a given metric was arbitrary. Preserving the sign of the correlations was particularly important in this study because we were interested in whether slow drift was associated with a pattern that is indicative of changes in the subjects’ arousal levels over time, i.e., decreased prestimulus α power, increased pupil size and decreased microsaccade rate. For simplicity, we adopted an identical approach to that used in our previous study (Johnston et al., 2021). That is, the sign of the slow drift was flipped if the majority of neurons had negative weights. Forcing the majority of neurons to have positive weights established a common reference frame in which an increase in the value of slow drift was associated with higher firing rates among the majority of neurons.

### Estimating the timescale of slow drift and prestimulus α power

To determine the timescale at which each variable fluctuated over the course of a session Gaussian smoothing was performed (Cowley et al., 2020). The first thing to note is that slow drift was computed in a slightly different manner. To improve temporal resolution, the 30-min sliding window (used to bin residual spike counts) was stepped every 1 min instead of every 6 min. The same approach was taken when computing prestimulus α power. Gaussian smoothing was then performed in a cross-validated manner using SDs (i.e., timescales) ranging from 1 to 90 min (step size = 1 min). To determine the timescale of the fluctuation, a R^2^ was computed for each SD by leaving out randomly chosen time points for each fold (10 in total) and then predicting the value of each held-out point by calculating a Gaussian weighted average of its neighbors. We found the SD with the maximum *R*^2^ and the SD at which the *R*^2^ dropped to 75% of the maximum *R*^2^. The latter was taken to be the timescale of the fluctuation.

### Choice of analysis time windows

We chose to analyze spiking responses during stimulus periods for consistency across studies. This was the approach taken in our original paper that discovered a slow drift of neural activity in macaque visual and prefrontal cortex (Cowley et al., 2020) and in a follow-up study that included other eye-related metrics such as microsaccade rate and evoked pupil size (Johnston et al., 2021). The reason we chose to analyze microsaccade rate during prestimulus periods was to avoid bi-phasic changes that occur during visual stimulus presentation. More specifically, microsaccade rate decreases shortly after a visual stimulus has been presented and then increases later (Engbert and Kliegl, 2003; Rolfs et al., 2008; Hafed and Ignashchenkova, 2013). With regards to segments of EEG data, decades of research has shown that decreased α power during prestimulus periods is associated with improved performance on change detection tasks (Ergenoglu et al., 2004; Babiloni et al., 2006; Hanslmayr et al., 2007; van Dijk et al., 2008; Busch et al., 2009; Mathewson et al., 2009; Romei et al., 2010). Therefore, we adopted the same approach and analyzed segments of EEG data during prestimulus periods.

### Data availability

All data and code for this manuscript are available at the following link: https://doi.org/10.1184/R1/19248827.

## Results

**Figure 2.**
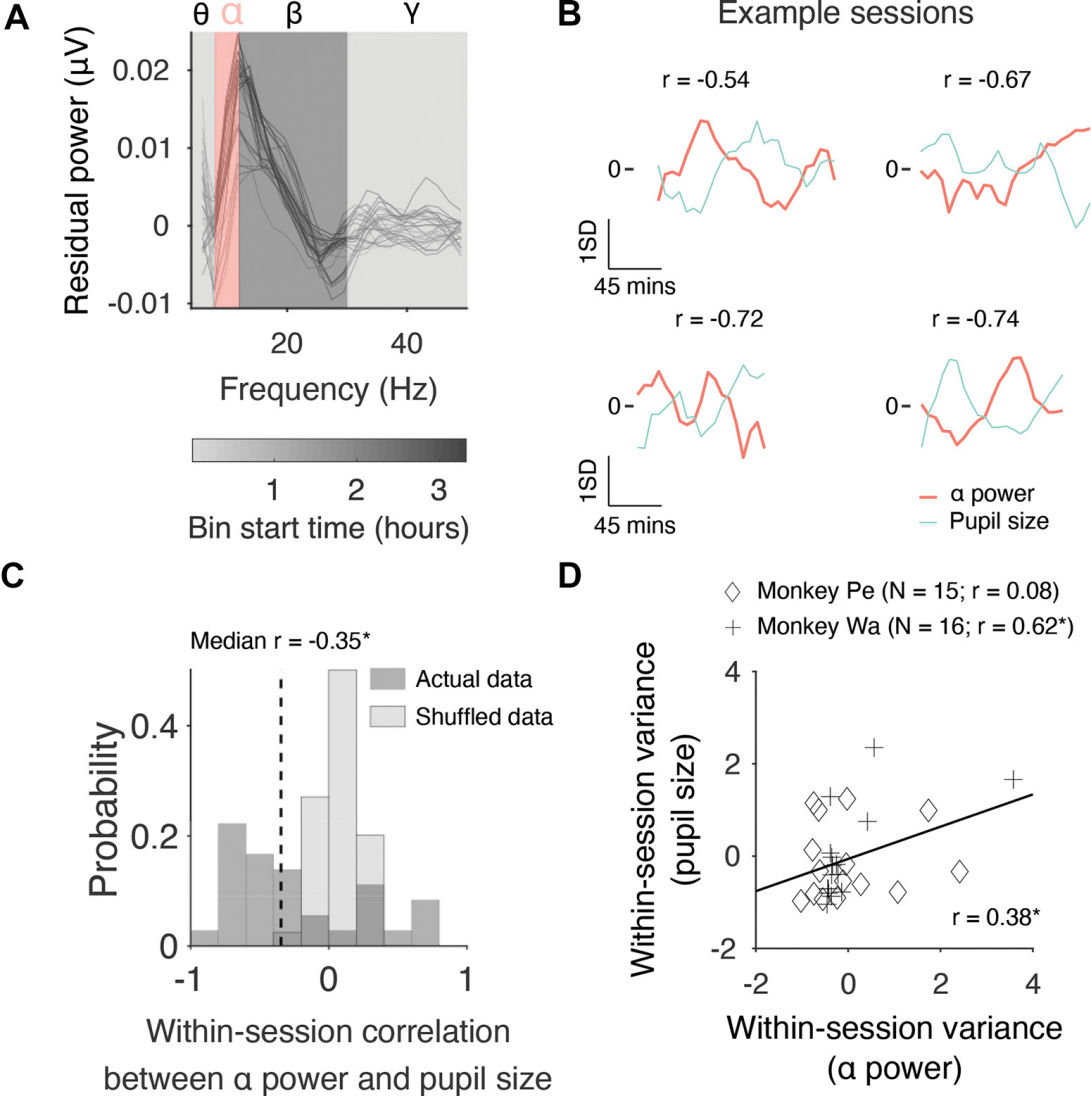
Correlation between prestimulus α power and raw pupil size. ***A***, Isolating slow fluctuations in prestimulus α power. A mean FFT was computed for each large 30-min bin (represented by gray lines) by averaging across electrodes. Note that the aperiodic portion of the signal was removed before averaging across electrodes (see Materials and Methods), which explains why residual power, as opposed to raw power, is shown on the *y*-axis. ***B***, Four example sessions in which there was a moderate to strong correlation between prestimulus α power and raw pupil size. The top row contains two sessions for Monkey Pe, whereas the bottom row contains two sessions for Monkey Wa. Note that the data were z-scored for visualization purposes only (i.e., so that variables with different units could be shown on the same plot). ***C***, A histogram showing the distribution of correlation values across sessions between prestimulus α power and raw pupil size. We used a Wilcoxon signed-rank test to test the null hypothesis that the median correlation across sessions was equal to zero (Monkey Pe: median *r* = −0.41, *p* = 0.008; Monkey Wa: median *r* = −0.31, *p* = 0.301). ***D***, A scatter plot showing how the magnitude of fluctuations in prestimulus α power (as measured by computing within-session variance) relate to the magnitude of fluctuations in raw pupil size. Note that the data were z-scored separately for each Monkey to control for potential differences in variance between subjects. Failing to control for this might have led to an artifactual correlation if the variance for one subject was greater than the other or vice versa. Individual correlations for each monkey have been reported in the figure legend along with the number of sessions included in the analysis; *p < 0.05, **p < 0.01, ***p < 0.001.

To determine whether spontaneous components of the EEG signal can provide insight into the internal brain state associated with slow drift, we trained two macaque monkeys to perform an orientation-change detection task in which pairs of stimuli were repeatedly presented (Fig. 1*A*). Spiking responses of populations of neurons in visual cortex (V4) were recorded using 100-channel “Utah” arrays as well as EEG on the scalp (Fig. 1*B*). For each electrode, FFTs were computed using segments of EEG data recorded during prestimulus periods. The data were then binned using a 30-min sliding window stepped every 6 min (Fig. 2*A*). Before averaging across electrodes, aperiodic activity that was 1/f-like in nature was estimated and subtracted off (see Materials and Methods). Finally, mean residual power was computed in distinct frequency bands. Our primary focus was on prestimulus α oscillations, but we also computed prestimulus power in the θ, β, and γ bands. In addition, raw pupil size and microsaccade rate were recorded during stimulus and prestimulus periods, respectively.

### Correlation between prestimulus α power and raw pupil size

First, we explored the relationship between prestimulus α power and raw pupil size. As described above, several studies in humans have established a link between these two variables using a combination of EEG and pupillometry (Hong et al., 2014; Van Kempen et al., 2019; Podvalny et al., 2021). Most recently, Compton et al. (2021) found that α power during intertrial periods on a Stroop task was inversely related to raw pupil size. That is, trials with greater pupil size were associated with reduced power in the α band and vice versa. To investigate whether a similar relationship was found in our data, raw pupil size measurements were binned using the same 30-min sliding window that was used to bin the EEG data. Note that the width of the window, and the step size, were chosen to isolate slow changes over time based on previous studies we performed (Cowley et al., 2020; Johnston et al., 2021). Example sessions for Monkey Pe and Monkey Wa are shown in Figure 2*B*, top and bottom rows, respectively. In support of previous research, prestimulus power in the α band was negatively associated with raw pupil size in each example session for both subjects.

The example sessions in Figure 2*B* exhibit moderate to strong trends. However, it is difficult to determine whether two variables that fluctuate slowly over time are correlated over the course of a single session. Standard correlation analyses assume that all samples are independent, but smoothed variables can violate this assumption leading to “nonsense correlations” between variables that are unrelated (Harris, 2020). An easy way to overcome this problem is to record data from multiple sessions. Two approaches can then be adopted: (1) one can compute a correlation for each session, and then perform a statistical test to investigate whether the distribution of coefficients across sessions is centered on zero; or (2) one can explore whether the magnitude of fluctuations in one of the variables is associated with the magnitude of fluctuations in the other variable. Both approaches were adopted in the present study to investigate whether there was a relationship between prestimulus α power and pupil size across sessions (Monkey Pe = 15 sessions; Monkey Wa = 16 sessions). First, we investigated whether prestimulus power in the α band and raw pupil size were correlated over time. Within each session, we computed a correlation (Pearson product-moment correlation coefficient) between prestimulus α power and raw pupil size. As in our previous study (Johnston et al., 2021), we found that null distributions (generated by computing correlations between sessions recorded on different days) were centered on zero. Therefore, a Wilcoxon signed-rank test was then used to test the null hypothesis that the median correlation across sessions was equal to zero. Consistent with the pattern of results observed in the four example sessions, we found that prestimulus α power was significantly and negatively correlated with raw pupil size (Fig. 2*C*, median *r* = −0.35, *p* = 0.012). Next, we investigated whether the magnitude of changes in EEG α power was correlated with the magnitude of changes in raw pupil size. Within each session, we computed the variance of prestimulus α power and raw pupil size. The data were then z-scored for each monkey separately to control for potential differences in variance that might have led to an artifactual correlation when the data were pooled across subjects. Finally, a correlation (Pearson product-moment correlation coefficient) was performed to investigate whether within-session variance in prestimulus α power was significantly associated with within-session variance in raw pupil size. Results showed that the magnitude of changes in prestimulus α power was significantly correlated with the magnitude of changes in raw pupil size (Fig. 2*D*, *r* = 0.38, *p* = 0.035). These findings support previous work in humans as they show that fluctuations in prestimulus α power were accompanied by global changes in the subjects’ arousal levels (Hong et al., 2014; Van Kempen et al., 2019; Compton et al., 2021; Podvalny et al., 2021). This motivated us to ask whether prestimulus α power is associated with other arousal-related metrics such as microsaccade rate.

### Correlation between prestimulus α power and microsaccade rate

**Figure 3.**
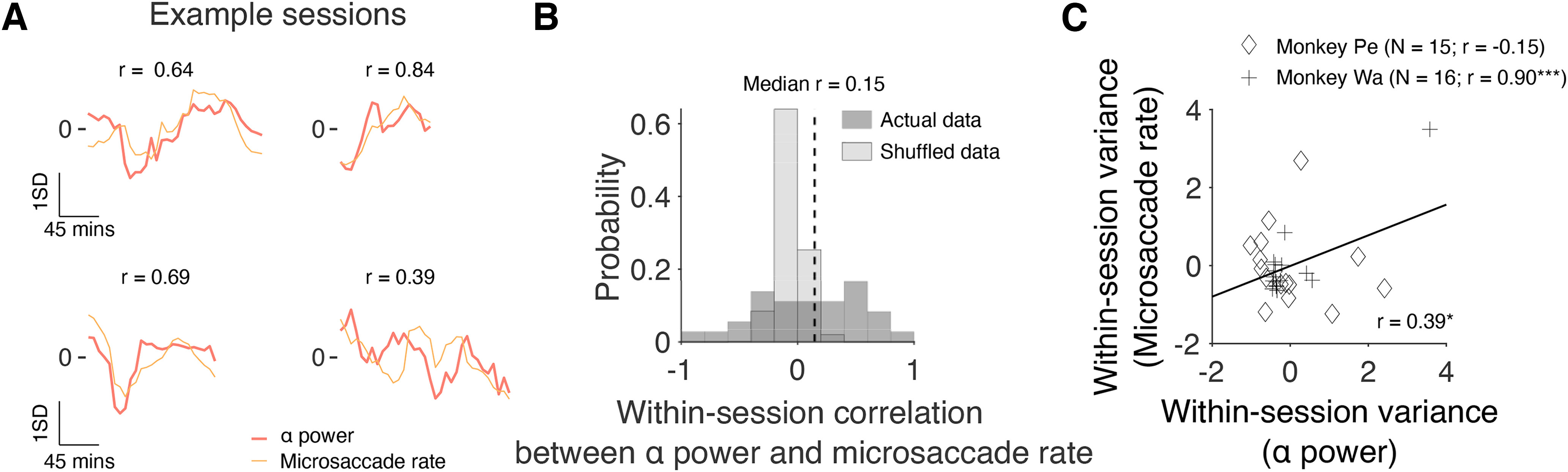
Correlation between prestimulus α power and microsaccade rate. ***A***, Four example sessions in which there was a moderate to strong correlation between prestimulus α power and microsaccade rate. The top row contains two sessions for Monkey Pe, whereas the bottom row contains two sessions for Monkey Wa. Note that the data were z-scored for visualization purposes only (i.e., so that variables with different units could be shown on the same plot). ***B***, A histogram showing the distribution of correlation values across sessions between prestimulus α power and microsaccade rate. A Wilcoxon signed-rank test was used to test the null hypothesis that the median correlation across sessions was equal to zero (Monkey Pe: median *r* = 0.23, *p* = 0.008; Monkey Wa: median *r* = −0.21, *p* = 0.44). ***C***, A scatter plot showing how the magnitude of fluctuations in prestimulus α power (as measured by computing within-session variance) relate to the magnitude of fluctuations in microsaccade rate. Note that the data were z-scored separately for each Monkey to control for potential differences in variance that might have resulted in an artifactual correlation. Individual correlations for each monkey have been reported in the figure legend along with the number of sessions included in the analysis; *p < 0.05, **p < 0.01, ***p < 0.001.

Recently, we found that microsaccade rate fluctuates over the course of a recording session in a manner that is consistent with slow changes in arousal over time (Johnston et al., 2021). More specifically, decreases in raw pupil size on a change detection task were accompanied by increases in microsaccade rate and vice versa. Hence, we were interested in whether prestimulus α power is correlated with microsaccade rate. Based on our previous research, we hypothesized that microsaccade rate would be positively correlated with prestimulus α power. To test this prediction, microsaccade rate measurements were binned using the same 30-min sliding window stepped every 6 min. Example sessions for Monkey Pe and Monkey Wa are shown in Figure 3*A*, top and bottom rows, respectively. In support of our hypothesis, prestimulus α power was positively associated with microsaccade rate in each example session for both subjects.

Next, we explored whether a similar pattern was present across all sessions (Monkey Pe = 15 sessions; Monkey Wa = 16 sessions). As before, we first investigated whether changes in prestimulus α power and microsaccade rate were correlated over time. Within each session, we computed the correlation (Pearson product-moment correlation coefficient) between prestimulus power in the α band and microsaccade rate. A Wilcoxon signed-rank test was then used to test the null hypothesis that the median correlation across sessions was equal to zero. Despite the compelling trends on some example sessions (Fig. 3*A*), there was no statistically significant correlation between prestimulus α power and microsaccade rate (Fig. 3*B*, median *r* = 0.15, *p* = 0.210). Next, we investigated whether the magnitude of changes in prestimulus α power was correlated with the magnitude of changes in microsaccade rate. Within each session, we computed the variance of prestimulus α power and microsaccade rate. A correlation (Pearson product-moment correlation coefficient) was then performed on z-scored data to investigate the relationship between within-session variance in prestimulus α power and within-session variance in microsaccade rate. Results showed that the magnitude of changes in prestimulus α power was significantly correlated with the magnitude of changes in microsaccade rate (Fig. 3*C*, *r* = 0.39, *p* = 0.029). Although we note that the median within-session correlation between slow drift and microsaccade rate was not significantly different from zero (Fig. 3*B*), the compelling match in the time course of individual sessions (Fig. 3*A*) and the significant session-by-session correlation in variance (Fig. 3*C*) are important for at least two reasons. First, they establish a novel link between prestimulus α power and fixational eye movements at long timescales. Second, they provide further evidence to suggest that fluctuations in prestimulus α power are associated with changes in arousal. If this is the case, one might expect α oscillations to be correlated with behavioral performance and other eye metrics that have been used to index changes in brain state such as saccadic reaction time and saccade velocity (Castellote et al., 2007; Di Stasi et al., 2013; DiGirolamo et al., 2016).

### Correlation between prestimulus α power and other arousal-related metrics

**Figure 4.**
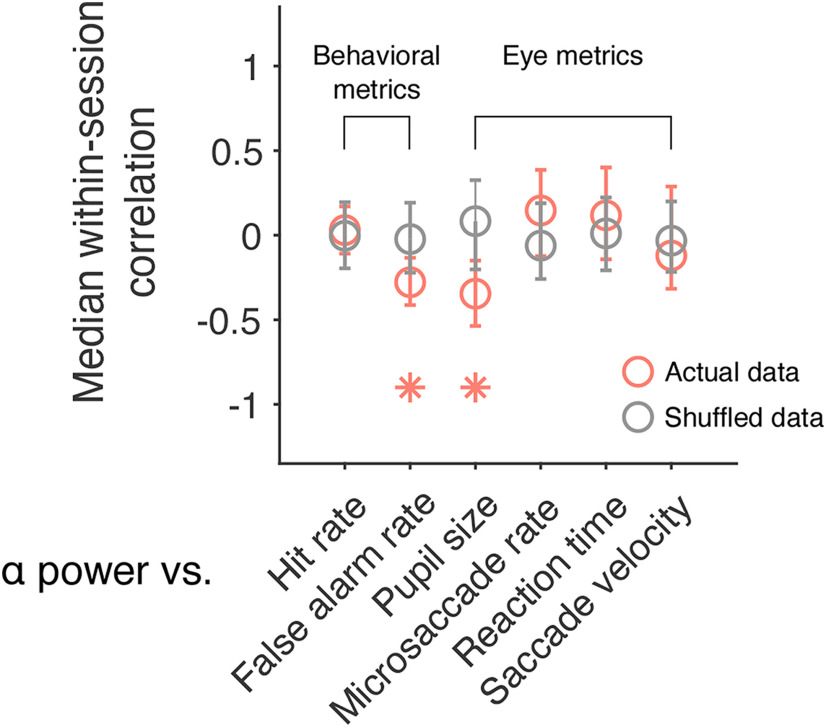
Correlation between slow drift and other arousal-related metrics. A scatter plot showing the median within-session correlation between prestimulus α power and a host of behavioral and eye metrics analyzed in our previous work (Cowley et al., 2020; Johnston et al., 2021). Note that pupil size and microsaccade rate have also been included so that comparisons can be made across the different behavioral and eye metrics. These data are the same as that reported in Figures 2*C* and 3*B*, respectively. For Monkey Pe, the median correlation between α power and false alarm rate was −0.28 (*p* = 0.083), whereas the median correlation between α power and pupil size was −0.41 (*p* = 0.008). For Monkey Wa, the median correlation between α power and false alarm rate was −0.24 (*p* = 0.326), whereas the median correlation between α power and pupil size was −0.31 (*p* = 0.301). Error bars represent bootstrapped 95% confidence intervals; *p < 0.05.

Previously, we found that performance on a change detection task, as measured by computing hit rate and false alarm rate, fluctuates slowly over the course of a recording session (Cowley et al., 2020). The same is also true of other eye metrics including saccadic reaction time and saccade velocity (Johnston et al., 2021). To explore whether prestimulus α power is associated with these additional arousal-related metrics, correlations (Pearson product-moment correlation coefficient) were computed within each session. Wilcoxon signed-rank tests were then used to test the null hypothesis that the median correlation across sessions was equal to zero. Results showed that prestimulus α power was negatively correlated with false alarm rate (Fig. 4, median *r* = −0.28, *p* = 0.046). However, it was not significantly correlated with hit rate (Fig. 4, median *r* = 0.03, *p* = 0.906), reaction time (median *r* = 0.12, *p* = 0.176) or saccade velocity (Fig. 4, median *r* = −0.12, *p* = 0.389). Note that pupil size and microsaccade rate have also been included in Figure 4 so that comparisons can be made across the different behavioral and eye metrics. These data are the same as that reported in Figures 2*C*, 3*B*, respectively. These findings are interesting because they demonstrate that prestimulus α power is associated with slow fluctuations in behavior over time. Previous research in humans has shown that prestimulus oscillations in the α band are associated with hit rate and false alarm rate (Iemi et al., 2017). However, it is difficult to make comparisons across studies because of task differences. In our paradigm, subjects had to make a saccade to the changed stimulus, whereas in Iemi et al. (2017), they had to make a yes/no decision about whether a near-threshold stimulus was presented. Either way, our results point to a link between α oscillations and global changes in arousal. Next, we asked whether prestimulus α power, a noninvasive signal recorded on the scalp, can be used to index a neural measure of brain state acquired directly from the spiking activity of neural populations.

### Correlation between prestimulus α power and slow drift

As described above, we recently observed a pervasive signal in visual and prefrontal cortex termed “slow drift” (Cowley et al., 2020). Interestingly, this neural signature was related to a subject’s tendency to make impulsive decisions on a change detection task and a constellation of eye metrics that are indicative of slow changes in arousal over time (Johnston et al., 2021). For example, we found that slow drift was positively correlated with raw pupil size and negatively correlated with microsaccade rate. This motivated us to ask whether noninvasive EEG signals recorded on the scalp are associated with slow drift. Based on our previous work, we hypothesized that prestimulus α power would be negatively correlated with slow drift.

**Figure 5.**
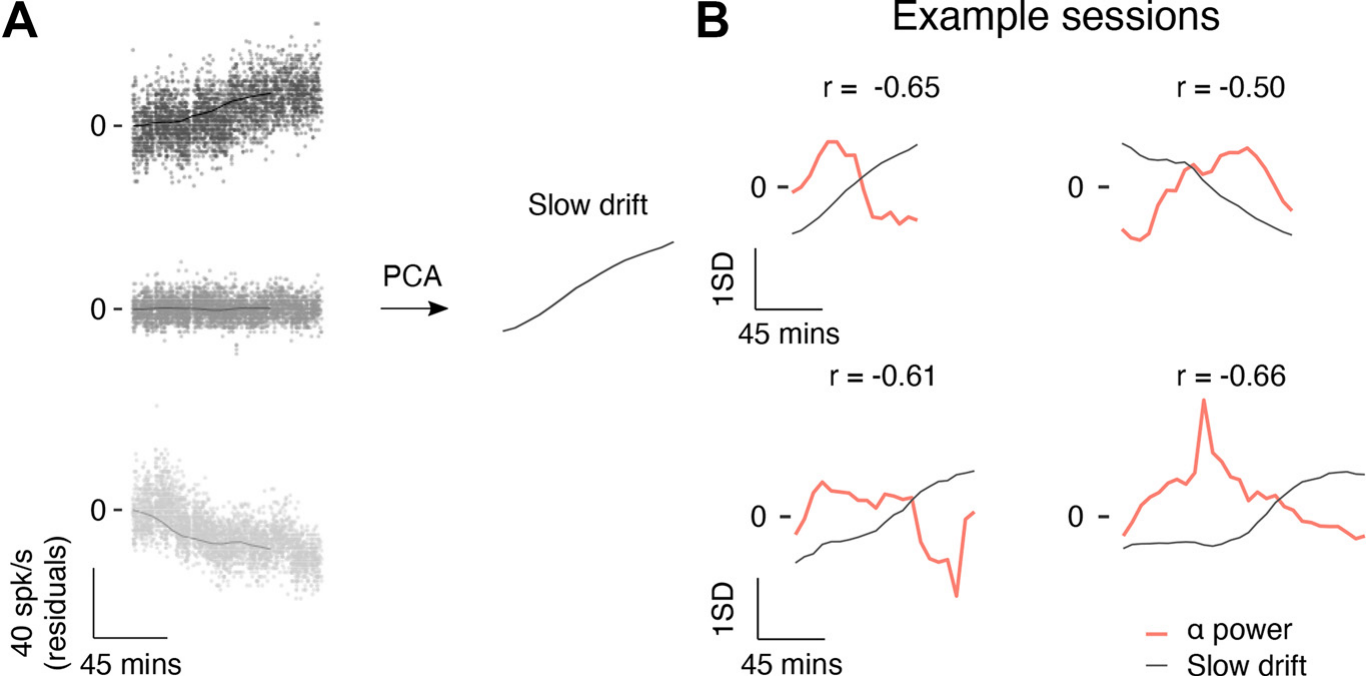
Computing slow drift. ***A***, Three example neurons recorded during the top left-hand session in ***B***. Each point represents the mean residual spike count during a 400-ms stimulus period. The data were then binned using a 30-min sliding window stepped every 6 min (solid line). PCA was used to reduce the dimensionality of the data and slow drift was computed by projecting binned residual spike counts along the first principal component. ***B***, Four example sessions in which there was a moderate to strong correlation between prestimulus α power and slow drift. The top row contains two sessions for Monkey Pe, whereas the bottom row contains two sessions for Monkey Wa. Note that the data were z-scored for visualization purposes only (i.e., so that variables with different units could be shown on the same plot). PCA, principal component analysis.

To calculate slow drift, residual spike counts were computed by subtracting the mean response for a given orientation across the entire session from individual responses. This was an important first step as it ensured that signals related to stimulus tuning were not present in the slow drift. Residual spike counts were then binned using the same 30-min sliding window that had been used to bin the EEG, pupil and microsaccade rate data (Fig. 5*A*; see Materials and Methods). We then applied PCA to the neural data and estimated slow drift by projecting the binned residual spike counts along the first principal component (i.e., the loading vector that explained the most variance in the data). The sign of the weights in PCA is arbitrary meaning that the correlation between slow drift and prestimulus α power in any session was equally likely to be positive or negative (Jolliffe and Cadima, 2016). To overcome this issue, we flipped the sign of the slow drift such that the majority of neurons had positive weights (Hennig et al., 2021). This established a common reference frame in which an increase in the value of the drift was associated with higher firing rates among the majority of neurons (see Materials and Methods).

We computed the slow drift of the neuronal population in each session using the above-mentioned method, and then compared it to prestimulus α power. Example sessions for Monkey Pe and Monkey Wa are shown in Figure 5*B*, top and bottom rows, respectively. In support of our hypothesis, prestimulus α power was negatively correlated with slow drift in each example session for both subjects.

**Figure 6.**
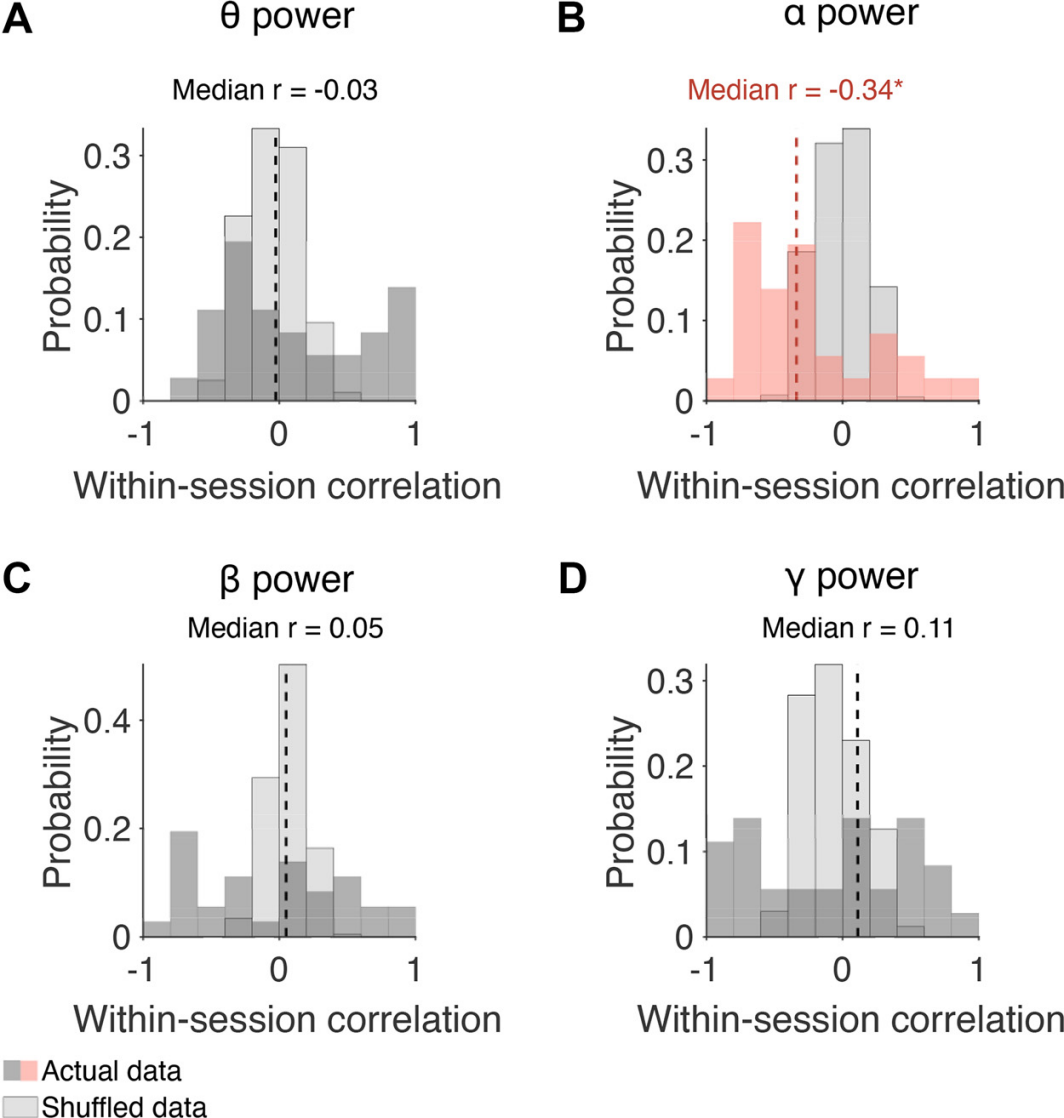
Correlation between slow drift and prestimulus power in distinct frequency bands. ***A–D***, Histograms showing distributions of correlation values across sessions. Wilcoxon signed-rank tests were used to test the null hypothesis that the median correlation across sessions was equal to zero. The median correlation between slow drift and α power for Monkey Pe was −0.47 (*p* = 0.041). For Monkey Wa, the median correlation between slow drift and α power was −0.24 (*p* = 0.215); *p < 0.05, **p < 0.01, ***p < 0.001.

Next, we explored whether a similar pattern was present across sessions (Monkey Pe = 15 sessions; Monkey Wa = 16 sessions). Within each session, a correlation (Pearson product-moment correlation coefficient) was computed between prestimulus α power and slow drift. Note that correlations were also performed to investigate the relationship between slow drift and prestimulus power in the θ, β, and γ bands. Wilcoxon signed-rank tests were then used to test the null hypothesis that the median correlation across sessions was equal to zero. Consistent with the pattern observed in the example sessions, prestimulus power in the α band was negatively correlated with slow drift (Fig. 6*B*, median *r* = −0.34, *p* = 0.017). No significant correlation was found between slow drift and prestimulus power in the θ (Fig. 6*A*, median *r* = −0.03, *p* = 0.318), β (Fig. 6*C*, median *r* = 0.05, *p* = 0.570), and γ (Fig. 6*D*, median *r* = 0.11, *p* = 0.531) bands. These findings suggest that spontaneous components of the EEG signal, namely prestimulus α power, recorded noninvasively on the scalp can be used to index low-dimensional patterns of neural activity acquired directly in the brain.

**Figure 7.**
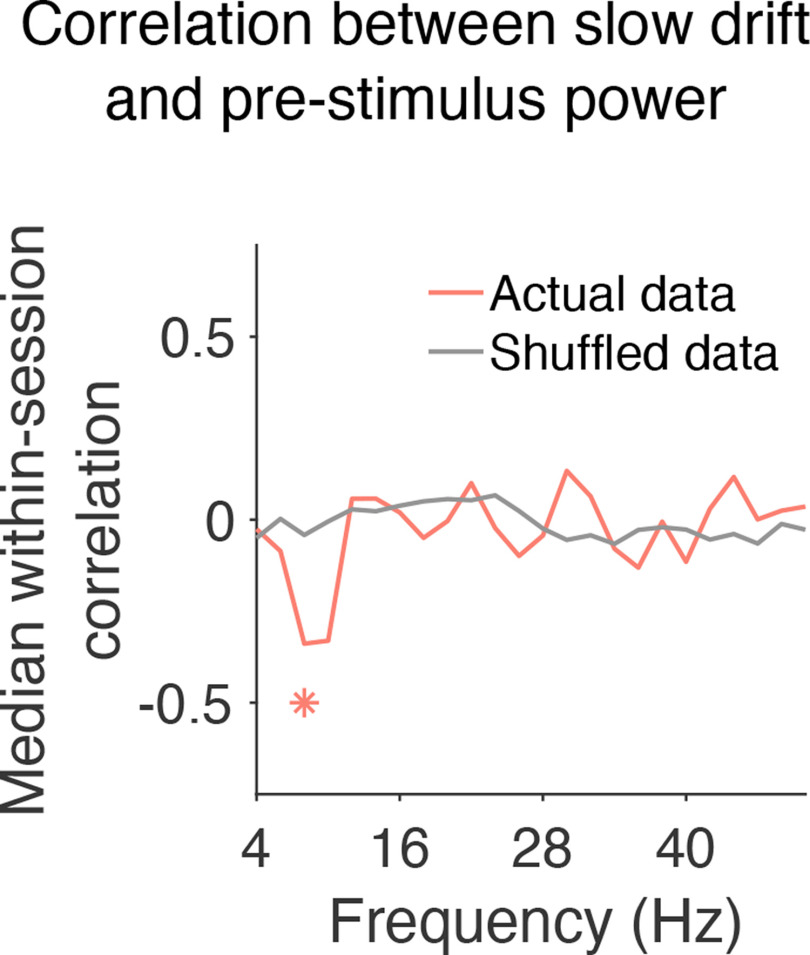
Correlation between slow drift and prestimulus power across frequencies. The median within-session correlation was computed using a 4-Hz sliding window stepped every 2 Hz. Values on the *x*-axis represent the left edge of the window. The only significant correlation was between slow drift and prestimulus power at 8–12 Hz, i.e., the α frequency band (*p* = 0.017). Note that the p-value for the correlation between slow drift and prestimulus power at 10–14 Hz was 0.06; *p < 0.05.

The frequency bands in the analysis described above (Fig. 6) were chosen based on previous research that has explored the relationship between EEG on the scalp and spiking responses recorded directly in the brain (Musall et al., 2014; Snyder et al., 2015). However, there are disadvantages to assessing power in predefined regions of the power spectrum such as variability in the peak central frequency that is known to be associated with different task demands and/or cognitive states (Mierau et al., 2017). Given our interest in the link between prestimulus oscillations and arousal, we performed an additional analysis to investigate whether slow drift was associated with power across a wide range of frequencies. To do this, we computed the median within-session correlation between slow drift and prestimulus α power using a 4-Hz sliding window stepped every 2 Hz. In support of the analysis described above (Fig. 6), which was performed using predefined frequency bands, we found that slow drift was only significantly correlated with prestimulus power at 8–12 Hz, the canonical α frequency band (Fig. 7, median *r* = −0.34, *p* = 0.017).

### Correlation between the timescale of slow drift and prestimulus α power

**Figure 8.**
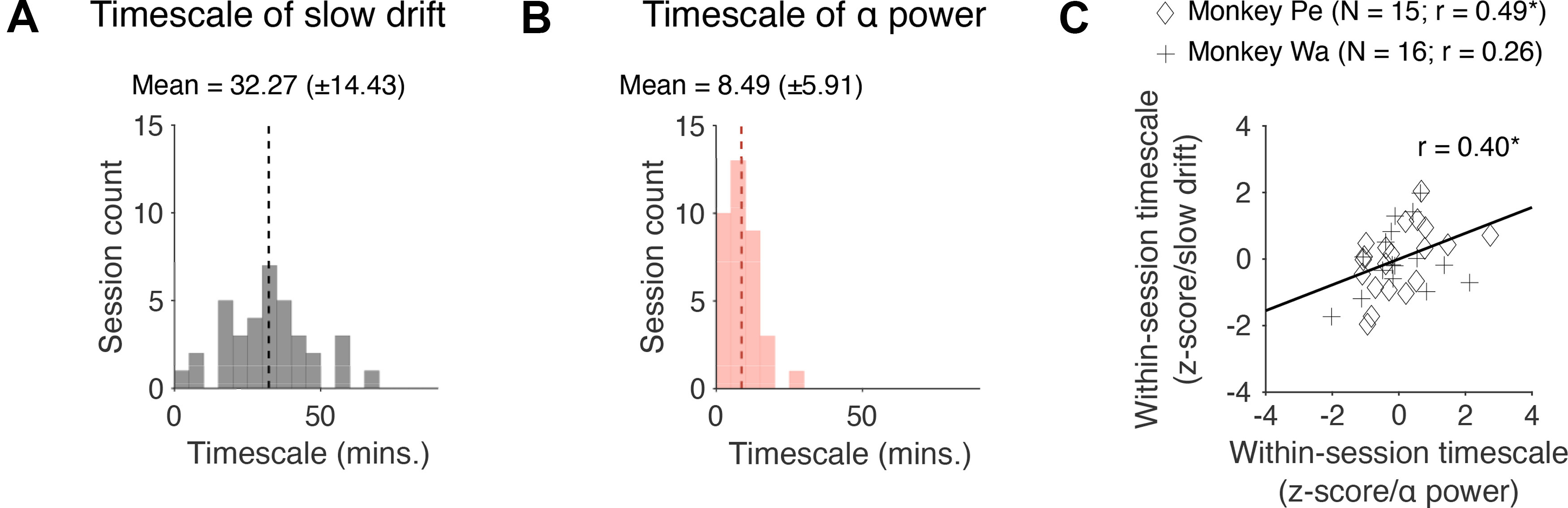
Correlation between the timescale of slow drift and prestimulus α power. ***A***, A histogram showing the distribution of timescales computed for slow drift across sessions. ***B***, Same as ***A*** but for prestimulus α power. ***C***, A scatter plot showing how the timescale of slow drift relates to the timescale of fluctuations in prestimulus α power. Note that the data were z-scored separately for each monkey to control for potential timescale differences between subjects. Failing to control for this might have led to an artifactual correlation if timescales for one subject were greater than those for the other or vice versa; *p < 0.05, **p < 0.01, ***p < 0.001.

Finally, we investigated whether the timescale of slow drift is correlated with the timescale of fluctuations in prestimulus α power. Note that these two variables are unlikely to have the same timescale because oscillations in the 8- to 12-Hz frequency band are influenced by cognitive processes such as attention that operate at a timescale of hundreds of milliseconds to seconds (Worden et al., 2000; Sauseng et al., 2005; Kelly et al., 2006; Snyder and Foxe, 2010). However, they may be modulated by a joint and continuously graded process (perhaps arising because of fluctuating levels of neuromodulators) that operates at a brain-wide level. If this is the case, one would expect a positive correlation between the timescale of slow drift and the timescale of fluctuations in prestimulus α power. To determine the optimal timescale at which a variable fluctuated over the course of a session, we used a cross-validated kernel regression method that applied Gaussian smoothing (with SDs ranging from 1 to 90 min) to the data and returned the time scale for smoothing that best fit held-out data (see Materials and Methods). We found that the timescale of slow drift (Fig. 8*A*, M = 32.27, SD = 14.43) was significantly longer than the timescale of fluctuations in prestimulus α power (Fig. 8*B*, M = 9.32, SD = 5.89; *t*_(65)_ = 8.28, *p* < 0.001). However, there was a significant correlation between the timescale of slow drift and prestimulus α power (*r* = 0.40, *p* = 0.0255) from session to session. This suggests that slow drift and prestimulus α power arise from distinct mechanisms on a moment-by-moment basis but may be linked to, or modulated by, a joint process that drives global changes in arousal over time.

## Discussion

In this study, we investigated whether prestimulus oscillations in the α band could be used as an external signature of an internal brain state, a recently discovered neural activity pattern called “slow drift” (Cowley et al., 2020). We know from previous work that slow drift in macaque visual and prefrontal cortex is correlated with a host of eye metrics across tasks with differing cognitive demands (Johnston et al., 2021), suggesting that slow drift can be used to index brain-wide changes in arousal. Since prestimulus α power is also related to arousal, we wondered whether a link could be established between a neural measure of an internal brain state acquired directly from the spiking activity of populations of neurons (i.e., slow drift) and indirect signals recorded noninvasively from the scalp using EEG. Results showed that slow drift was significantly associated with a pattern that is indicative of changes in the subjects’ arousal levels over time: decreases in prestimulus α power were accompanied by increases in raw pupil size and decreases in microsaccade rate.

Several studies in humans have found a relationship between prestimulus α power and pupil size (Hong et al., 2014; Van Kempen et al., 2019; Podvalny et al., 2021). For example, on classic Stroop tasks, trials with greater raw pupil size are associated with reduced α power and vice versa (Compton et al., 2021). Our results support this finding as slow changes in prestimulus α power were negatively correlated with raw pupil size. Furthermore, there was an association between the magnitude of fluctuations in prestimulus α power (as measured by computing within-session variance) and the magnitude of fluctuations in raw pupil size. These findings suggest that prestimulus α power is associated with global changes in brain state that occur naturally over time, perhaps because of fluctuating levels of neuromodulators in the brain. Testing this hypothesis would require the simultaneous recording of EEG from the scalp and spiking activity in subcortical regions associated with arousal such as the LC (Aston-Jones and Cohen, 2005; Sara, 2009; Chandler, 2016). Such studies have been conducted in monkeys (Foote et al., 1980; Swick et al., 1994) but the relationship between prestimulus α power and pupil size has not been elucidated in awake behaving animals performing cognitively demanding tasks. Hence, an important question for future research would be to determine how the spiking activity of subcortical regions associated with arousal relates to prestimulus α power recorded on the scalp.

Another noninvasive metric that has been linked to oscillations in the α band is microsaccade rate. We know from previous research that visual perception is altered up to 700 ms after a microsaccade has occurred, at a frequency of ∼8–20 Hz (Bellet et al., 2017). The results of the present study show that a relationship also exists between α oscillations and microsaccade rate at longer timescales. Recently, we found that slow fluctuations in raw pupil size were negatively correlated with microsaccade rate (Johnston et al., 2021). That is, microsaccade rate decreased under conditions of heightened arousal (as indexed by greater pupil size and increased saccade velocity) and vice versa. Given this result, we hypothesized that there would be a positive correlation between prestimulus α power and microsaccade rate at longer timescales, which are more likely to reflect changes in a subject’s internal cognitive state (Cowley et al., 2020). This is exactly what was found in several example sessions for both monkeys. Furthermore, the magnitude of fluctuations in prestimulus α power was significantly correlated with the magnitude of fluctuations in microsaccade rate across sessions. A key goal for future research is to bridge the gap between microsaccade-related EEG signals and neural activity in brain regions that have been implicated in eye movement control such as the SC (Martinez-Conde et al., 2013). Research combining EEG and eye tracking has shown that microsaccades are accompanied by: (1) a large potential over occipital electrodes ∼100 ms after movement onset; and (2) changes in α/θ power (Dimigen et al., 2009). However, it is unclear how these signals recorded on the scalp, at relatively short timescales, relate to the activity of SC neurons. Similarly, we do not know how long timescale changes in prestimulus α power, which we found to be correlated with microsaccade rate, link to firing rates in the SC. Future research combining eye tracking, EEG and single unit/population recordings in the SC is needed to determine the neural underpinnings of noninvasive scalp signals that are associated with fixational eye movements.

Taken together, the results of the pupil size and microsaccade rate analysis suggest that fluctuations in prestimulus α power are associated with global changes in arousal. This motivated us to ask whether noninvasive signals recorded on the scalp can be used to index a recently discovered signature of an internal brain state called “slow drift” (Cowley et al., 2020). We know from previous research that slow drift is positively correlated with raw pupil size and negatively correlated with microsaccade rate across tasks with differing cognitive demands (Johnston et al., 2021). Therefore, we predicted that there would be an inverse relationship between changes in prestimulus α power and slow drift over time. In support of this hypothesis, we found that slow drift in visual cortex was negatively correlated with prestimulus α power in several example sessions for both monkeys. Furthermore, they were significantly and negatively correlated when the data were pooled across sessions. It is important to note that this finding cannot be attributed to changes in 1/f noise that are characteristic of several brain disorders (Peterson et al., 2017; Robertson et al., 2019) and healthy aging (Voytek et al., 2015). First, aperiodic components of the FFT were estimated and subtracted off before averaging across electrodes (Donoghue et al., 2020). Second, no significant correlation was found between slow drift and prestimulus power in the θ, β, and γ bands. One might have expected this to be the case if slow drift was associated with uniform shifts in power across frequencies. Nonetheless, 1/f noise and other aperiodic fluctuations, might be related to global brain modulations in ways that are not well captured by the slow drift we observe. A further interesting question is whether slow drift is associated with other EEG signals such as the P1 component of the visually evoked potential (VEP). There are at least two reasons why this might be the case. First, evidence suggests that early VEP components are negatively correlated with prestimulus α power (Roberts et al., 2014; Iemi et al., 2019). Second, decades of research has shown that P1 amplitude is associated with global changes in brain state (Luck et al., 2000). For example, it is significantly larger on trials in which a weak visual stimulus is detected (Ergenoglu et al., 2004; Pourtois et al., 2006; Del Cul et al., 2007; Mathewson et al., 2009) and negatively correlated with reaction time on spatial attention tasks (Mangun and Hillyard, 1991).

Another key finding from the present study is related to the timescale of slow drift and fluctuations in prestimulus α power. We found that the timescale of these two variables was significantly different: prestimulus α power had a shorter timescale than slow drift. This result is unsurprising given that oscillations in the 8- to 12-Hz frequency band are influenced by processes such as attention that operate at a timescale of hundreds of milliseconds to seconds (Worden et al., 2000; Sauseng et al., 2005; Kelly et al., 2006; Snyder and Foxe, 2010). However, we did find that the timescale of slow drift and the timescale of fluctuations in prestimulus α power was significantly correlated across sessions. This observation is important because it suggests that these two variables are modulated by a common process such as arousal that operates at a brain-wide level. In the present study, there was no experimental manipulation of the subjects’ arousal levels meaning that the timescale of slow drift and prestimulus α power varied naturally from session to session. Evidence suggests that LC is a major source of fluctuations in arousal (Aston-Jones and Cohen, 2005; Sara, 2009; Chandler, 2016). Therefore, activating neurons in this region directly via electrical microstimulation, or indirectly via pharmacological manipulations, should lead to correlated changes in the timescale of slow drift and prestimulus α power.

In summary, we found that a commonly used metric of cognitive state in human EEG studies, prestimulus α power, is associated with gradual shifts in the underlying population structure of neural activity throughout the brain. Together, these measures at the scalp, and in the cortex, were predictive of changes in the monkeys’ arousal levels over time. These findings show that indirect measures of neural activity can be used to index a global signature of arousal. By linking a vast EEG literature in humans with simultaneous scalp/microelectrode array recordings in macaques our results bridge the gap between large-scale field potentials and the spiking responses of populations of cortical neurons.

## Synthesis

Reviewing Editor: Mark Laubach, American University Decisions are customarily a result of the Reviewing Editor and the peer reviewers coming together and discussing their recommendations until a consensus is reached. When revisions are invited, a fact-based synthesis statement explaining their decision and outlining what is needed to prepare a revision will be listed below. The following reviewer(s) agreed to reveal their identity: Charles Wilson, Anne Urai. Note: If this manuscript was transferred from JNeurosci and a decision was made to accept the manuscript without peer review, a brief statement to this effect will instead be what is listed below.

Two expert reviewers reviewed your study. Their reviews are given below. Please address all points that were raised.

In discussing your study in our review forum, it was strongly felt that you should share the data and code from your study on sites such as OSF. Also Reviewer 1 wrote that while their proposed changes were framed as “optional though preferable”, it was strongly felt that you should include the suggested behavioural analyses.

Reviewer 1

Johnston et al present results from simultaneous EEG and single unit recordings in macaques performing an orientation change detection task. The group have previously presented evidence for a “slow drift” phenomenon in non-stimulus driven spiking, that is proposed to index arousal of subjects. The aim here was to determine whether a minimally invasive approach like EEG could index this phenomenon, and they provide some correlative evidence that pre-stimulus alpha power in the 8-12Hz range does do this, and that in turn alpha fluctuates with both raw pupil size and microsaccade rate, both implicated in indexing arousal.

This manuscript has been reviewed at the Journal of Neuroscience (not my me), and I have received reviews, although curiously not the decision letter that means it has come to eNeuro. The authors appeared to have addressed those comments pretty well, significantly revised the manuscript, and removed a contentious part of the data, apparently a VEP analysis. Not knowing why the manuscript was subsequently rejected (I would guess some spurious argument about impact), I can’t really comment on whether the exclusionary factor for J Neuro is also prejudicial for eNeuro.

Taken on face value the manuscript is well written, the aims and analyses clear, and the results of interest to the community. The effort to link single cell responses, necessarily acquired by invasive recordings, to non-invasive EEG signals is a worthwhile (and valiant) one, and it’s something we don’t do enough in the neurophysiology community. In already making a revision the authors have addressed several concerns I would otherwise have had. This work should be published.

Arousal is of course a slippery customer in formal analytic terms, as many features thought to index arousal are going to be modulated by many other task and non-task features. As such I think it is important for authors to supply all the information that might potentially support a claim about arousal, and here that should include the behaviour as well as microsaccades and pupil size. In fact the authors have specifically removed the analyses linking alpha power to behaviour, as a way of dealing with a previous reviewer comment. I think that regardless of what they show, these results are important features of the data, and should be included in any publication. And by the way I think the authors’ response to reviewer 2 (point 10) on the validity of these analyses is justified.

This point is particularly important given the lack of manipulations of arousal, also referred to by a previous reviewer. In a sense the authors have a natural experiment of arousal manipulation, in that monkey arousal will vary between the sessions, but also within a session in a manner that it might be possible to infer from behaviour. Given that the key claim here (in my eyes) linking alpha and slow drift (Fig 5) is based on a series of session-by-session correlations, how behaviour varies between sessions that do or do not show a significant correlation is a crucial analysis that is lacking here I suggest it be included regardless of the result.

More generally, a statistical modelling approach that brings together the various repeated measures recorded across sessions (alpha, drift, microsaccades, pupil size, cognitive behaviour) might be useful to allow us to understand the size of the effects presented here, and their size relative to each other, in a way that the current approach (session-by-session correlation followed by Wilcoxon test) does not. There is a variety of statistical approaches (starting with some mixed-effect or structural-equation models) that I think would strengthen this manuscript and bring the findings together. I would consider this to be desirable but not essential for publication.

Reviewer 2

In this paper, Johnston et al. use a unique dataset of concurrent spiking activity, scalp EEG, behavior and various eye-tracking and pupil measurements. They have previously shown that slow drifts in spiking activity can capture behaviorally relevant fluctuations (which one may call ‘arousal’ or ‘impulsivity’), and also linked this signal to pupillometry and microsaccades. Here, they tap into a previously unreported subset of the data in which concurrent EEG was recorded, and show that power in an 8-10 Hz band correlates with this neural slow drift.

Since this paper has already undergone a prior round of review in which I did not participate, I hope to refrain from making too many further suggestions. The work clearly contributes to our understanding of the many ways in which arousal could be quantified, spanning across species. The use of methods to correct for nonsense correlations, as described by Harris, is an important one and I applaud the authors for taking this into account.

I do have some important suggestions for clarification, and improving the further use of these data for the field as a whole.

Most importantly, I strongly encourage the authors to make these data and analysis code publicly available (e.g. https://crcns.org/). Over and above increasing the transparency and reproducibility of scientific findings as a whole, sharing data and code increases the visibility and impact of individual papers (McKiernan et al. 2016 eLife). While I appreciate that there is not yet a widespread culture of data sharing in NHP research, I believe that this dataset could be a fantastic ground truth for more quantitatively testing the link between scalp EEG and neural firing (e.g. Cohen, TiN 2017). The data has already been repeatedly analyzed by this group, and sharing can further will help bridge the large gap between many researchers working on arousal and EEG in humans, with the systems neuroscience community.

How were the frequency bands chosen (l. 242-244)? Figure 2a (and the original bioRxiv Figure 2A too) do not show a clear oscillatory peak at the selected bands, perhaps due to human-monkey differences. The methods proposed by Donoghue, which the authors use to remove the 1/f component, also proposed data-drive methods to identify real oscillatory bands on top of the 1/f (which I currently do not see clearly captured in the frequency band delineation). Another way to address this frequency-specificity would to add a panel to Figure 5 with the median (+ IQR) correlation (+ significance from shuffled data) per frequency bin in steps of e.g. 1 Hz, rather than sticking with these pre-defined bands that may not match the monkey oscillatory peaks.

Relatedly, I find it curious that the aperiodic component is removed the 1/f slope and offset could itself by interesting targets for arousal-related signals. I don’t expect the authors to dive into this now, but if the data were shared this could be a great follow-up. It could also be mentioned that the same findings hold for unadjusted power (as in the bioRxiv figure 5).

I find it rather confusing that the timewindows differ between some of the metrics (pre-stimulus or post-stimulus). What is the rationale for that? Wouldn’t it make more sense to extract all metrics from the pre-stimulus window to ensure maximal comparability (this would also remove the need to correct for orientation tuning in V4 responses)? It would be interesting to hear if the authors think any such preprocessing differences may contribute to the timecourse differences in figure 6.

Dealing with nested dependencies (e.g. sessions within a monkey) is not trivial (Aarts et al. 2014 NN), and I’m unsure if z-scoring per monkey before doing a correlation removes all confounds. Could the authors also report a correlation coefficient for each monkey separately, in figures 2D and 3C?

Around l. 440, the authors’ conclusions are too strong: they do not find an effect across sessions, but then proceed to interpret the data as if an effect was there. Please tone this down.

I have two suggestions for visualization. The first would be to add the shuffled distributions back in, as in the bioRxiv version of the paper (I find them very informative, and I’m not sure why they are absent from this new version). Second, it would hugely help the me (and presumably other working-memory-taxed readers) to see a schematic overview figure with the known and novel correlations between all these putative measures of ‘arousal’. By now there are quite a few: slow drift, EEG bands, pupil, microsaccade, behavior (d’/c), all with their own paired positive/negative correlations. The authors nicely review relevant findings from the literature in both humans and NHPs: if this could be shown in e.g. a diagram with boxes and arrows or a heatmap correlation matrix (indicating which studies tested what relationship and in which species), it would be immediately clear which of the empty boxes is filled by the current work.

I find the link to behavior very relevant, and I’m disappointed that the authors have decided to take it out. Perhaps they could further speculate on ways that these different neural, pupil and saccadic measures may drive behavior (see e.g. de Gee et al. 2020 eLife for model-based analyses of behavior across species), especially given their work in the Cowley paper.
